# Supplementary material for: Induction of muscle stem cell quiescence by the secreted niche factor Oncostatin M
Source: Nat Commun. 2018 Apr 18;9:1531. doi: 10.1038/s41467-018-03876-8 (PMC5906564; doi:10.1038/s41467-018-03876-8)
Supplement: Supplementary file 1 — Supplementary Information [file 41467_2018_3876_MOESM1_ESM.pdf]

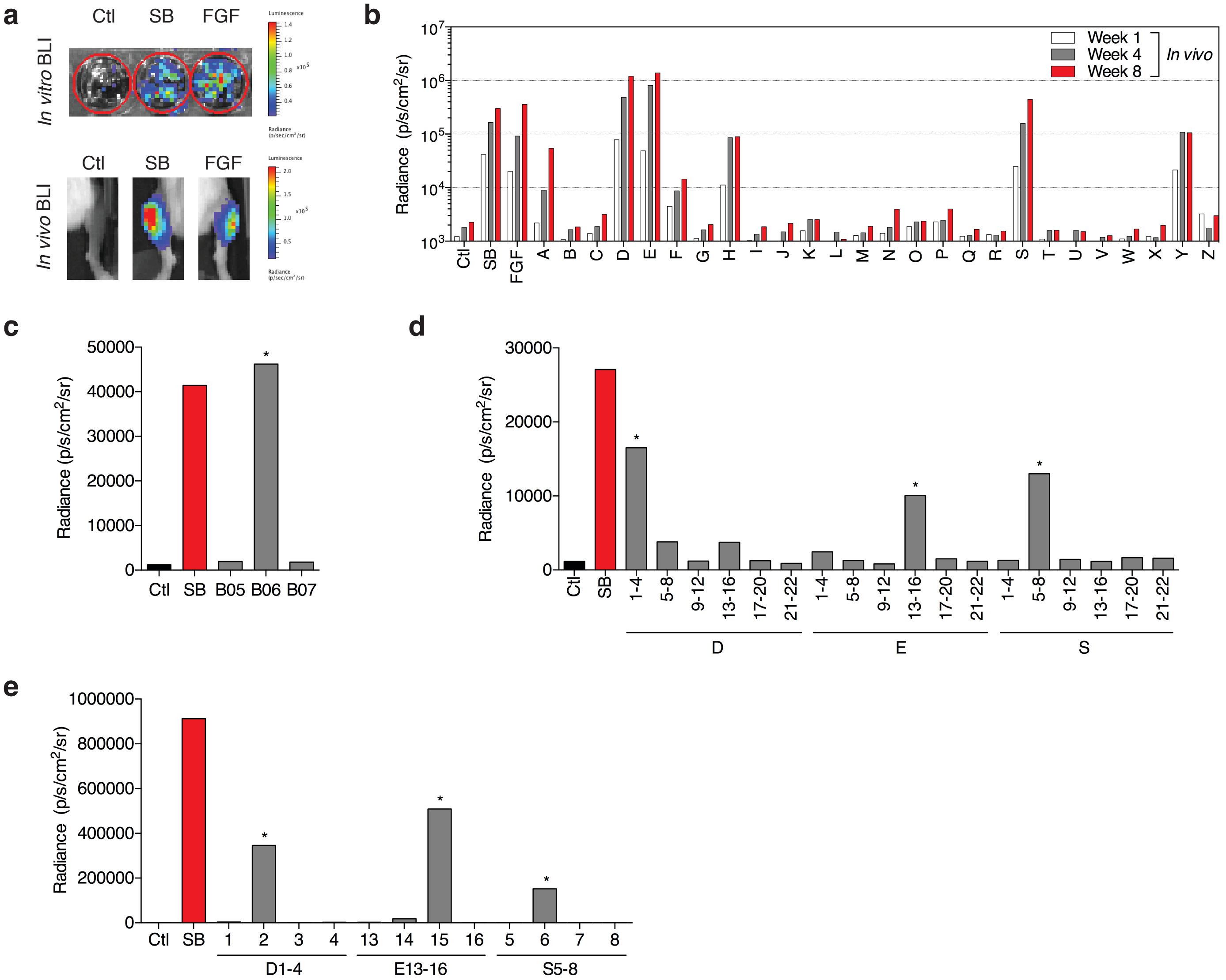

Supplementary Figure 1

Deconvolution of screening hits.

(a) Representative *in* vitro and *in vivo* BLI results for the screening strategy. 800 FACS-sorted MuSC were cultured on plastic in minimal conditions either without additional factors (Ctl), with SB202190 (SB; 10 µm), or with recombinant human Fibroblast Growth Factor (FGF; 2.5 ng/mL). *In vitro* BLI was performed after 6 days, the cell were then harvested and then transferred intramuscularly. *In vivo* BLI was performed 2 weeks later.

(b) Serial *in vivo* BLI from multiplexed protein screening. BLI was performed at 1 (white), 4 (grey), and 8 (red) weeks post transplant. Ctl: control buffer; SB: SB202190; FGF: recombinant human basic Fibroblast Growth Factor.

(c) *In vivo* BLI data from final round of deconvolution of pool Y (see Fig. 1c). SB202190 (SB; 10 µm), and buffer (Ctl) were used as controls. Asterisk indicates final positive pool, containing the extracellular domain of TNFRSF1A.

(d) *In vivo* BLI data from deconvolution of three positive pools from Fig. 1c, each containing 4 proteins. SB202190 (SB; 10 µm), and buffer (Ctl) were used as controls. Imaging was performed at day 10 following intramuscular transplant. Asterisk indicates positive subpools.

(e) *In vivo* BLI data from final round of deconvolution of positive subpools indicated in (c). Note that each subpool now contains an individual protein. Imaging was performed at day 14 following intramuscular transplant. Asterisk indicates positive subpools, each containing Oncostatin M.

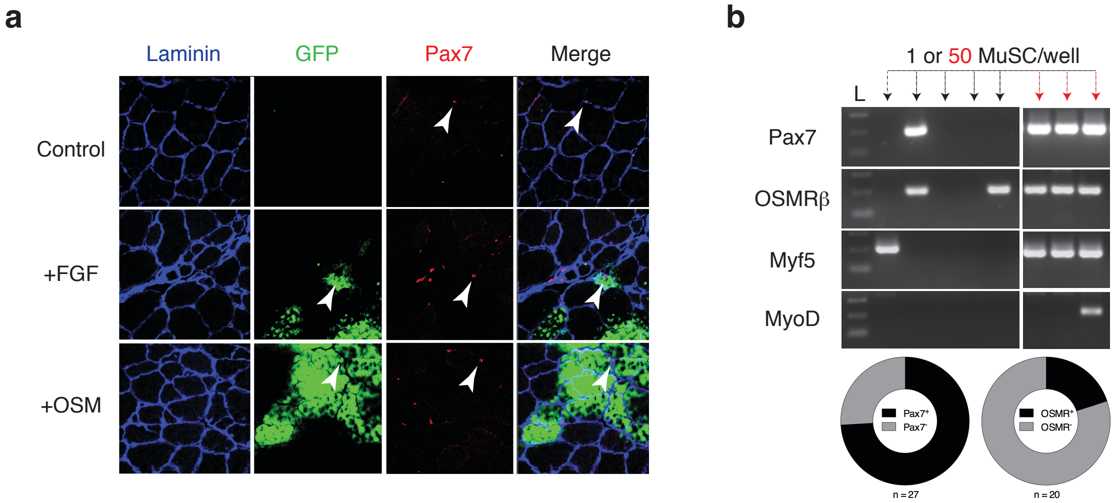

Supplementary Figure 2

Expression of OSMRβ within muscle stem cells.

(a) Representative immunofluorescence analysis of muscle sections following transplant with either buffer (top row), FGF (middle row) or OSM (bottom row) treated stem cells isolated from CAG-*luciferase-GFP* mice. Note that engrafted fibers derived from these cells express both luciferase as well as GFP. White arrowheads indicate $Pax7^+$ satellite cells adjacent to areas of GFP^+ donor-derived fibers.

(b) OSMRβ expression on single sorted MuSC. Triple FACS-sorted MuSCs were sorted into PCR tubes at either 1 cell per well or 50 cells per well (positive control) for RT-PCR. Representative data from nested, non-quantitative RT-PCR demonstrating expression of Pax7, OSMRβ, Myf5, and MyoD in either single cells (black arrows) or in pools of 50 cells (red arrows). Of cells demonstrating amplification, $OSM^+/Pax7^+$ was ~19% (20 total), $OSM^+Myf5^+/Myf5^+$ was ~13% (13 total), $Pax7^+$ was 75% (27 total), $Myf5^+$ was ~48% (13 total). Schematic at bottom summarizes Pax7 and OSMRβ results.

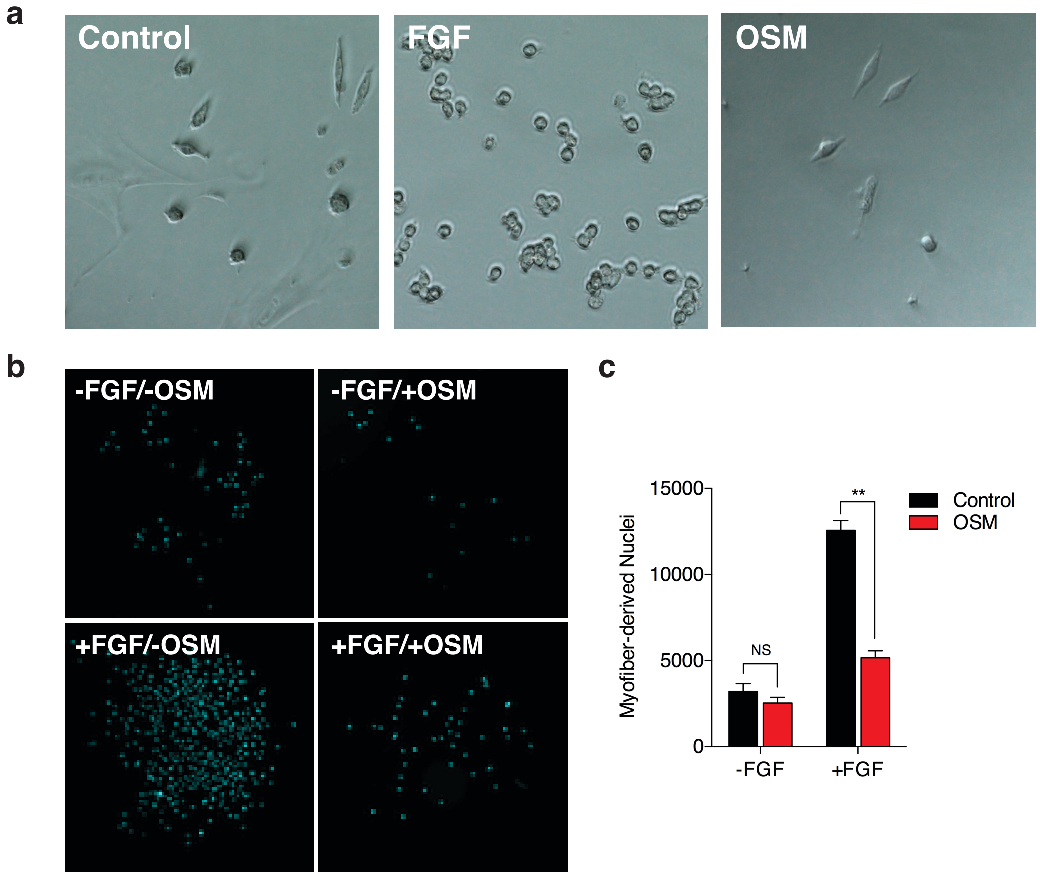

Supplementary Figure 3

OSM represses proliferation of isolated and fiber-associated muscle stem cells.

(a) Phase contrast micrograph images of FACS-sorted MuSC culture for 6 days with either control buffer (left), FGF (2.5 ng/ml; middle), or OSM (100 ng/ml; right). Images were acquired at 20X.

(b) Imaging of cells derived from myofiber cultures described in Fig. 3c. Bulk muscle fibers derived from C57BL/6 mice were cultured for 5 days in the simultaneous absence or presence of FGF and/or OSM. Plate-bound cells were quantified by DAPI staining, high-content imaging, and automated counting of cell nuclei.

(c) Quantification of myofiber-derived colony formation. Bulk muscle fibers from wild type C57BL/6 mice were cultured for 5 days in the absence or presence of FGF and OSM. At the end of the culture period, plate-bound cells representing progeny of activated satellite cells were quantified by high-content imaging and automated counting of $DAPI^+$ nuclei (see Supplementary Fig. 3b). NS, not significant; double asterisk indicates $p<0.01$.

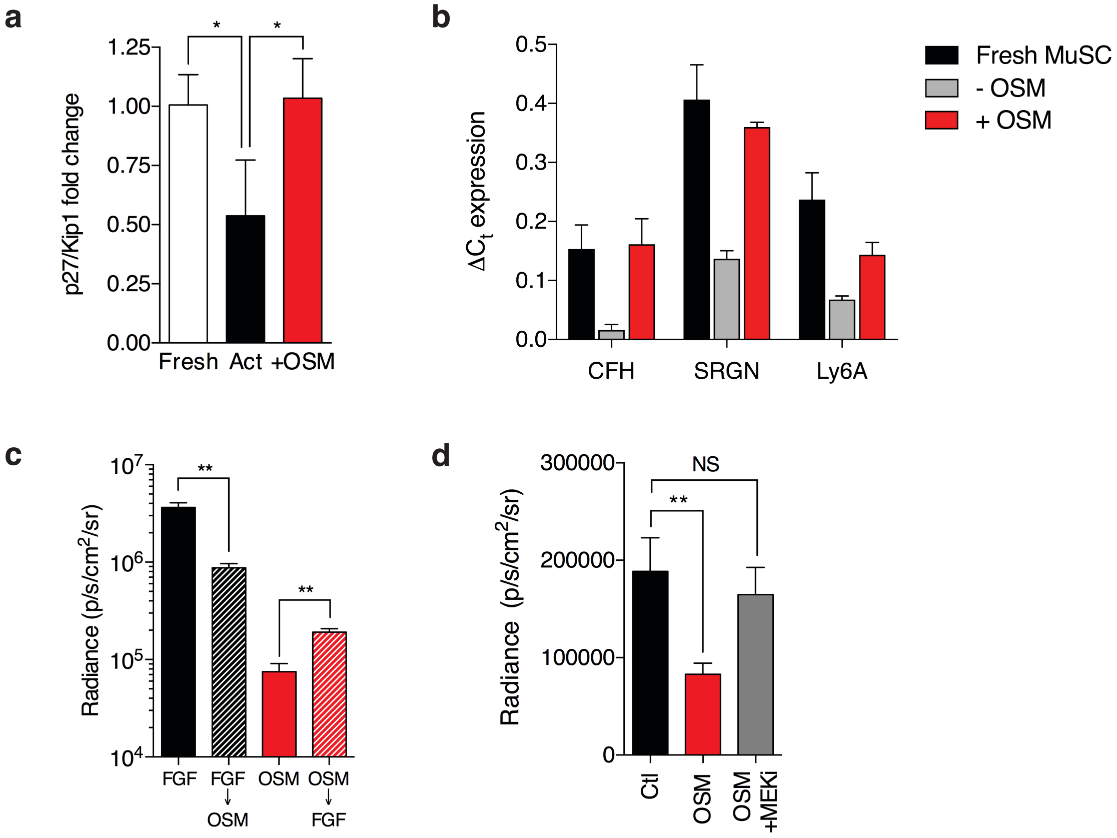

Supplementary Figure 4

Mechanism of OSM-induced quiescence.

(a) p27/Kip1 induction in response to OSM treatment *in vitro*. qPCR was performed on FACS-sorted MuSC either freshly isolated (Fresh) or cultured for 6d in the absence (Activated, Act) or presence (+OSM) of OSM. Data were normalized to expression of GAPDH. Mean +/- SD is shown, n=3 replicates; asterisk indicates $p<0.05$.

(b) Validation of quiescence gene signature. Expression of three genes from the microarray-derived quiescence signature was independently assessed by qPCR analysis of fresh MuSC or MuSC cultured without (-OSM) or with OSM (+OSM).

(c) *In vitro* BLI for FACS-sorted MuSC from Fig. 2e. Cells were cultured for 6 days under the indicated conditions, and BLI was performed prior to cell fixation. Mean +/- SD is shown, n=4 replicates.

(d) Proliferation of FACS-sorted MuSC cultured for 6 days in the presence or absence of OSM, or with both OSM and the MEK inhibitor U0126 (5 μM). Mean +/- SD is shown, n=3 replicates.

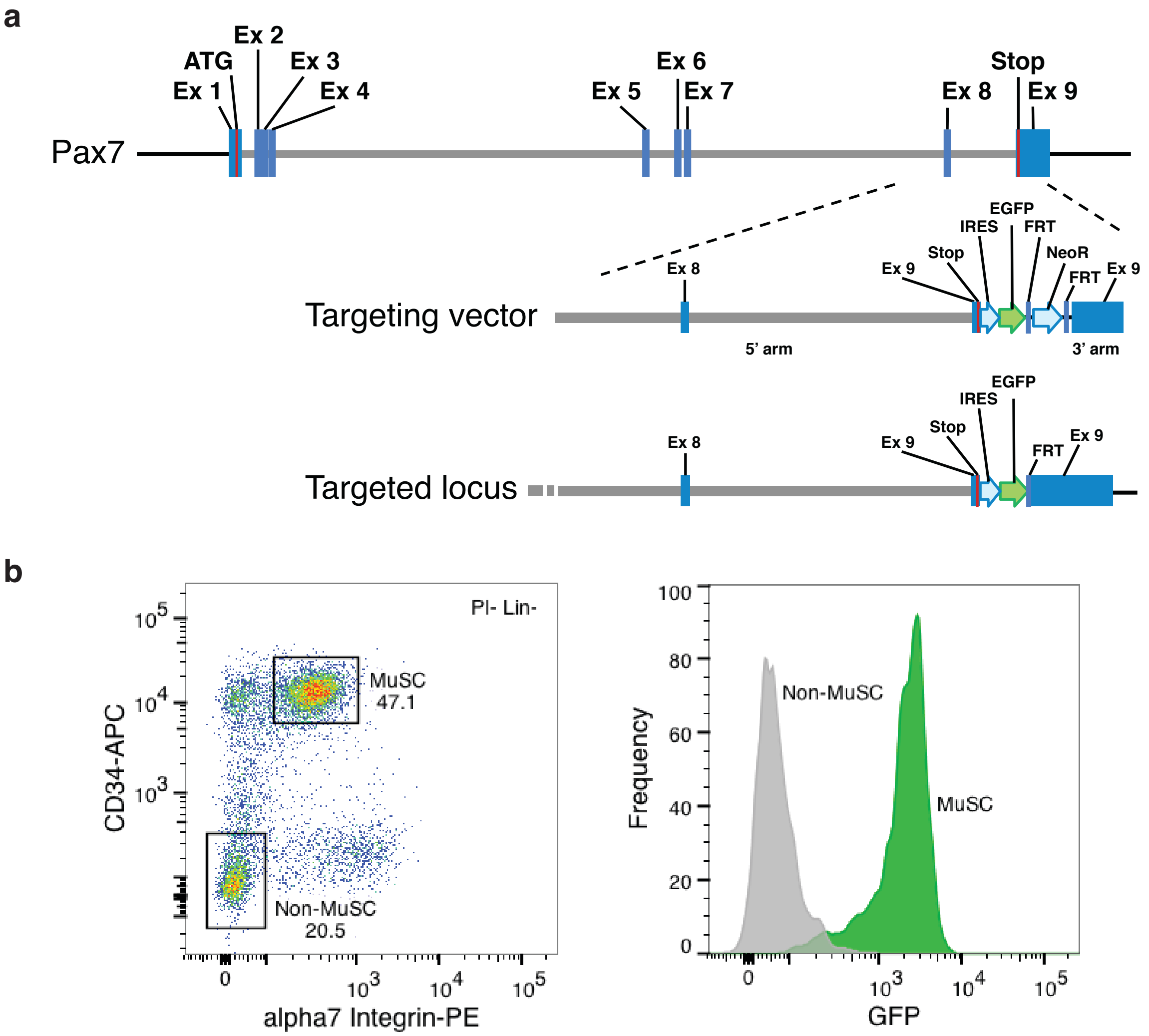

Supplementary Figure 5

Generation and validation of Pax7^{GFP} allele.

(a) Schematic of targeting vector used to generate Pax7^{GFP} mice, containing insertion of an Internal Ribosome Entry Site and Green Fluorescent Protein (IRES-GFP) cassette into the 3' untranslated region of the *Pax7* locus.

(b) FACS analysis of the muscle-derived mononuclear fraction from homozygous Pax7^{GFP} mice. Left: Gating of PI$^-$CD11b$^-$Sca1$^-$CD31$^-$CD45 cells into MuSC and non-MuSC populations by CD34 and α7-Integrin expression. Right: GFP expression in MuSC (PI$^-$CD11b$^-$Sca1$^-$CD31$^-$CD45$^-$CD34$^+$α7 integrin$^+$; green) and non-MuSC (PI$^-$CD11b$^-$Sca1$^-$CD31$^-$CD45$^-$CD34$^+$α7 integrin$^-$; grey) populations.

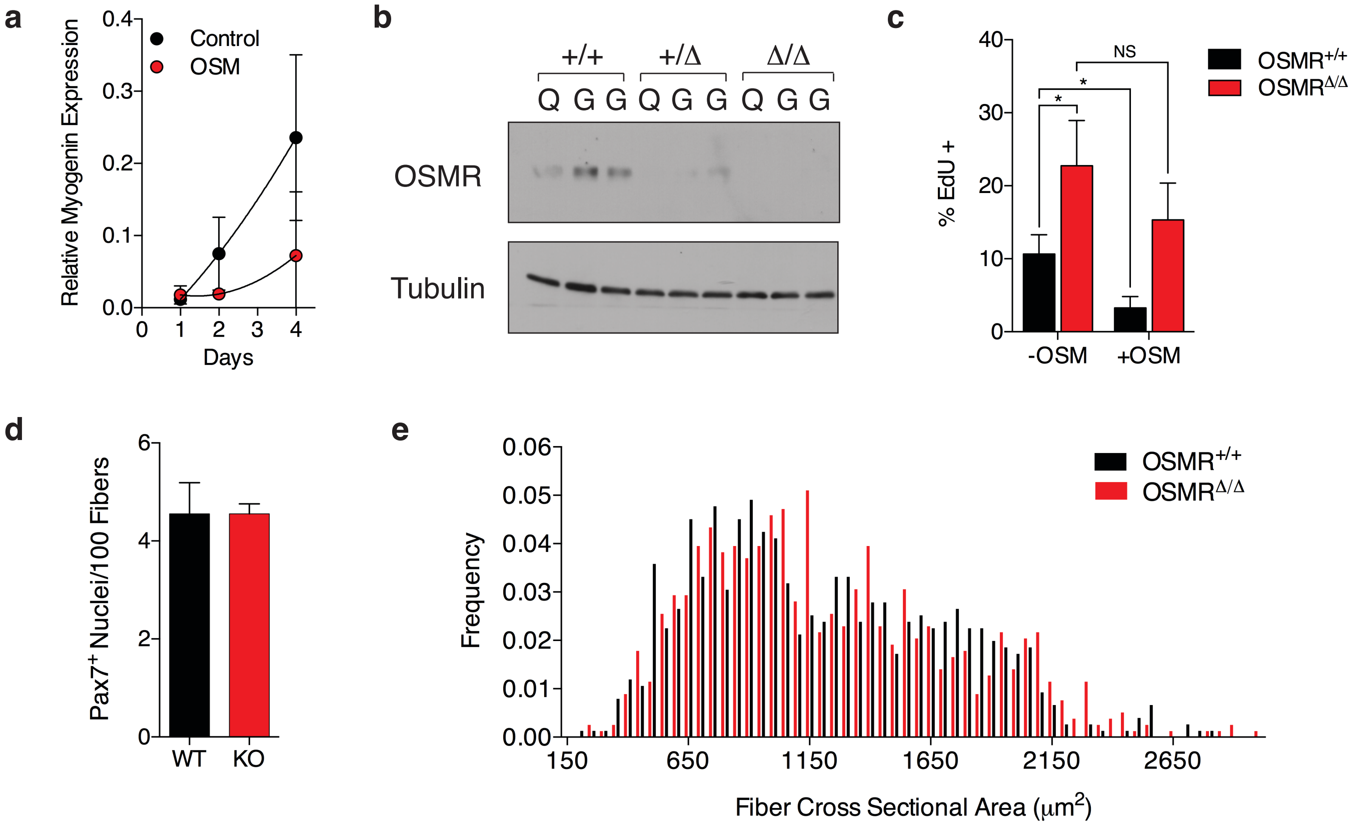

Supplementary Figure 6
OSMR is required for OSM signaling in muscle stem cells.
(a) qRT-PCR analysis of *myogenin* expression following 6 day culture of FACS-sorted MuSC in the presence of OSM (gray circles) or buffer control (black). Mean +/- SD is shown, n=3 replicates.
(b) Western blot analysis of uninjured quadriceps (Q) or gastrocnemius (G) muscle from wild type (+/+), heterozygous (Δ/+) or homozygous (Δ/Δ) mutant OSMRβ animals.
(c) Increased proliferation of OSMRβ-deficient MuSC. FACS-sorted wild type ($OSMR^{+/+}$) or homozygous OSMRβ mutant ($OSMR^{Δ/Δ}$) muscle stem cells were cultured for 6 days without (-OSM) or with OSM (+OSM). Cells were pulsed labeled with EdU for the final 12 hours of culture.
(d) Quantitation of $Pax7^+$ stem cells in serial sections from tibialis anterior muscle of uninjured wild type (+/+) or homozygous (Δ/Δ) mutant OSMRβ animals. Results are expressed relative to fiber number.
(e) Cross-sectional area (CSA) distribution of myofibers from tibialis anterior (TA) muscle of uninjured wild type (+/+; open boxes) or homozygous (Δ/Δ; black boxes) mutant OSMRβ animals. >750 fibers were quantified per genotype.

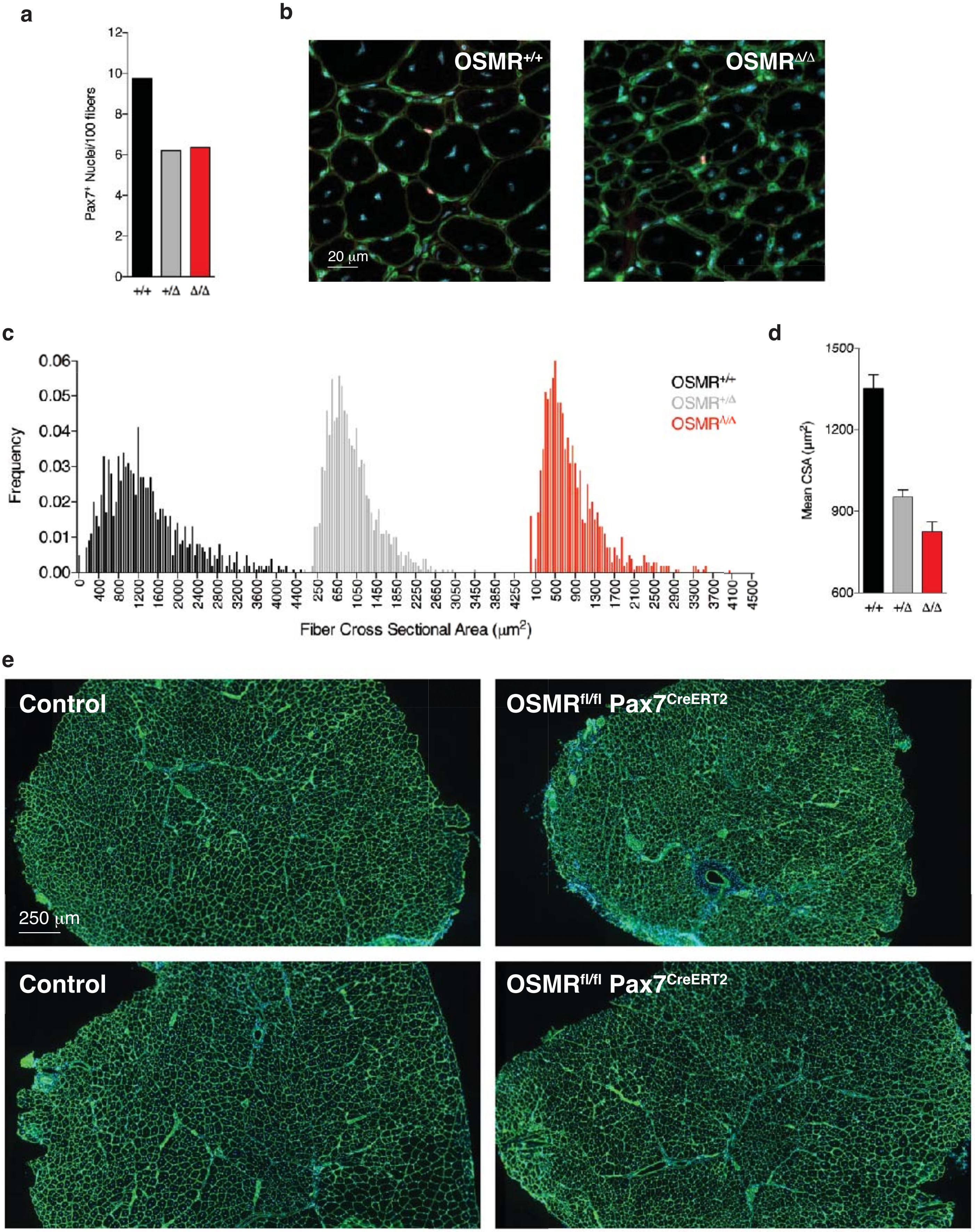
a
b
c
d
e
Pax7+ Nuclei/100 fibers
+/+
+/Δ
Δ/Δ
OSMR+/+
OSMRΔ/Δ
20 μm
Frequency
OSMR+/+
OSMR+/Δ
OSMRΔ/Δ
Fiber Cross Sectional Area (μm2)
Mean CSA (μm2)
Control
OSMRfl/fl Pax7CreERT2
250 μm

Supplementary Figure 7

Characterization of muscle regeneration in OSMRβ deficient mice.

(a) Quantitation of Pax7^{+} stem cells in serial sections from tibialis anterior muscle of wild type (+/+), heterozygous (Δ/+) or homozygous (Δ/Δ) mutant OSMRβ animals following 2 rounds of cardiotoxin injury and regeneration. Results are expressed relative to fiber number.

(b) Representative immunofluorescence images from tibialis anterior muscle of wild type (+/+) or homozygous mutant (Δ/Δ) OSMRβ animals following 2 rounds of cardiotoxin injury and regeneration. Blue: DAPI; Green: Laminin; Red: Pax7.

(c) Cross-sectional area (CSA) distribution of myofibers from tibialis anterior (TA) muscle of wild type (+/+; open boxes), heterozygous (Δ/+; gray boxes) or homozygous (Δ/Δ; black boxes) mutant OSMRβ animals following 2 rounds of cardiotoxin injury and regeneration. >1000 fibers were quantified per pooled genotype.

(d) Quantification of mean CSA from TA muscle section described in (e). >1000 fibers were quantified per pooled genotype. 95% confidence intervals of the mean are indicated.

(e) Low magnification images of immunofluorescence images from tibialis anterior muscle of control (Osmr$^{fl/+}$) or conditional knockout (Osmr$^{fl/fl}$ Pax7^{CreERT2}) animals following deletion and 2 rounds of cardiotoxin injury and regeneration. Blue: DAPI; Green: Laminin.

Supplementary References

1 Fukada, S. *et al.* Molecular signature of quiescent satellite cells in adult skeletal muscle. *Stem cells* **25**, 2448-2459, doi:10.1634/stemcells.2007-0019 (2007).

2 Pallafacchina, G. *et al.* An adult tissue-specific stem cell in its niche: a gene profiling analysis of in vivo quiescent and activated muscle satellite cells. *Stem cell research* **4**, 77-91, doi:10.1016/j.scr.2009.10.003 (2010).
