# Supplementary material for: Induction of muscle stem cell quiescence by the secreted niche factor Oncostatin M
Source: Nat Commun. 2018 Apr 18;9:1531. doi: 10.1038/s41467-018-03876-8 (PMC5906564; doi:10.1038/s41467-018-03876-8)
Supplement: Supplementary file 2 — Description of Additional Supplementary Files [file 41467_2018_3876_MOESM2_ESM.pdf]

Description of Additional Supplementary Files

File Name: Supplementary Data 1

Description: A transcriptional signature of satellite cell quiescence. Genes showing up- or down-regulation between quiescent and activated satellite cells was compared between two literature datasets1,2 as well as microarray analysis of satellite cells cultured in vitro for 3 days with or without Oncostatin M. The top 10% of genes demonstrating regulation in all 3 datasets is shown, totaling 613 genes. Note the presence of expected OSM transcriptional targets, including Osmr and Socs3. Q1, A1 refer to quiescent and activated cells from dataset 1; Q2, A2 refer to quiescent and activated cells from dataset 2.
